# Megalosplenia as an initial manifestation of multiple myeloma with a novel CYLD gene mutation: A case report and literature review

**DOI:** 10.1097/MD.0000000000037624

**Published:** 2024-04-05

**Authors:** Jinjing Zhang, Rui Zhang

**Affiliations:** a Department of Hematology, the First Affiliated Hospital of China Medical University, Shenyang, Liaoning 110001, China

**Keywords:** bendamustine, CYLD, daratumumab, multiple myeloma, spleen plasmacytoma, t(14;16)

## Abstract

**Introduction:**

Megalosplenia in newly diagnosed multiple myeloma (MM) is extremely rare, posing diagnostic and therapeutic challenges due to its unusual location and clinical manifestations and lack of optimal therapeutic strategies.

**Case presentation:**

A 65-year-old female who was previously healthy presented with a history of ecchymosis on her right leg accompanied by progressive fatigue for 2 weeks. She was admitted to our center in July 2019 due to thrombocytopenia. The patient presented with megalosplenia, anemia, monoclonal protein (λ-light chain type) in the serum and urine, and 45.6% malignant plasma cells in the bone marrow. Splenectomy was performed due to persistent splenomegaly after 3 cycles of the bortezomib plus dexamethasone regimen, and immunohistochemistry results indicated λ-plasmacytoma of the spleen. The same cytogenetic and molecular abnormalities, including t(14;16), 14q32 amplification, 16q32 amplification, 20q12 amplification, and a novel CYLD gene mutation, were identified using fluorescence in situ hybridization and next-generation sequencing in both bone marrow and spleen samples. Therefore, a diagnosis of MM (λ-light chain type, DS III, ISS III, R-ISS III, high-risk) with spleen infiltration was proposed. The patient did not achieve remission after induction treatment with bortezomib plus lenalidomide and dexamethasone or salvage therapy with daratumumab plus ixazomib and dexamethasone. However, she ultimately did achieve very good partial remission with a regimen of bendamustine plus lenalidomide and dexamethasone. Unfortunately, she died of pneumonia associated with chemotherapy.

**Conclusion:**

To our knowledge, only 8 cases of spleen plasmacytoma at MM diagnosis have been described previously. Extramedullary myeloma patients with spleen involvement at diagnosis are younger and that the condition is usually accompanied by splenic rupture with aggressive clinical features and poor prognosis. Further studies are needed to explore pathogenesis and effective therapies to prolong the survival of such patients.

## 1. Introduction

Multiple myeloma (MM) is the second most common hematologic malignancy caused by clonal plasma cell proliferation; it is usually characterized by the presence of malignant plasma cells in the bone marrow accompanied by MM-defining events including hypercalcemia, renal failure, anemia, and lytic bone lesions.^[1]^ Although in most MM patients, clonal plasma cells are restricted to the bone marrow, myeloma cells can spread into other organs or tissues, which is called extramedullary myeloma (EMM). EMM includes extramedullary myeloma extraosseous (EME), which involves the soft tissue or viscera at extraosseous locations, and extramedullary myeloma bone-related, including paraskeletal infiltration originating from the extension of bone disease.^[2]^ According to the largest retrospective study evaluating a total of 3744 MM patients, the incidence of EME at diagnosis is only 3.7%.^[3]^ In particular, spleen involvement in MM at the time of initial diagnosis is extremely rare, and to our knowledge, as few as 8 cases have been published in the literature to date.^[4–11]^ Most of these cases were reported as splenic rupture secondary to MM, with overall survival <1 year or even several days.^[5–7]^ Herein, we describe an unusual case of spleen infiltration in a newly diagnosed MM patient whose initial manifestation was megalosplenia, an atypical manifestation of MM. In addition, we review published cases concerning newly diagnosed MM with spleen involvement. The main purpose of the present case study was to describe the clinical features, examinations and treatment of spleen infiltration in newly diagnosed MM, aiming to improve our understanding of this rare but potentially fatal complication of MM.

## 2. Case presentation

**Figure 1.**
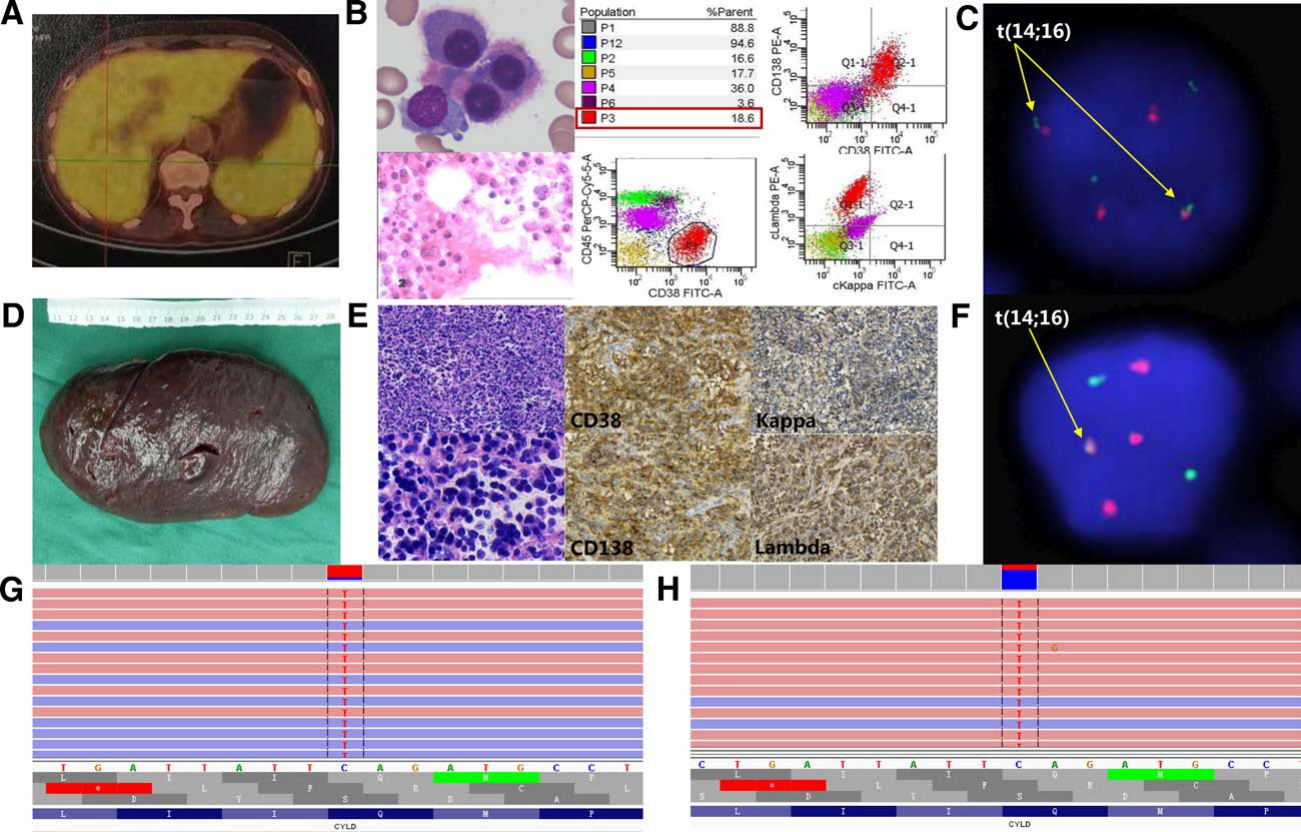
The imaging, bone marrow and spleen examinations of the patient. (A) Enlarged liver and spleen with increased FDG uptake (SUV of 5.3) revealed by PET-CT. (B) Malignant plasma cells in bone marrow detected by morphology, FCI and biopsy. These PCs mainly expressed CD38, CD138, cLambda and were negative for cKappa. (C) Rearrangement and amplification of the MAF gene (IgH/MAF Dual-Color Dual Fusion probe) of BM. (D) The resected spleen was as large as 19 × 10 × 5.5 cm. (E) Diffuse infiltration of malignant plasma cells in the spleen. (F) Rearrangement and amplification of the MAF gene (IgH/MAF Dual-Color Dual Fusion probe) of spleen. (G) and (H) A novel point mutation of CYLD (NM_015247: exon17: c. C226 3T: p.Q755X) of BM (G) and spleen (H) discovered by NGS.

**Figure 2.**
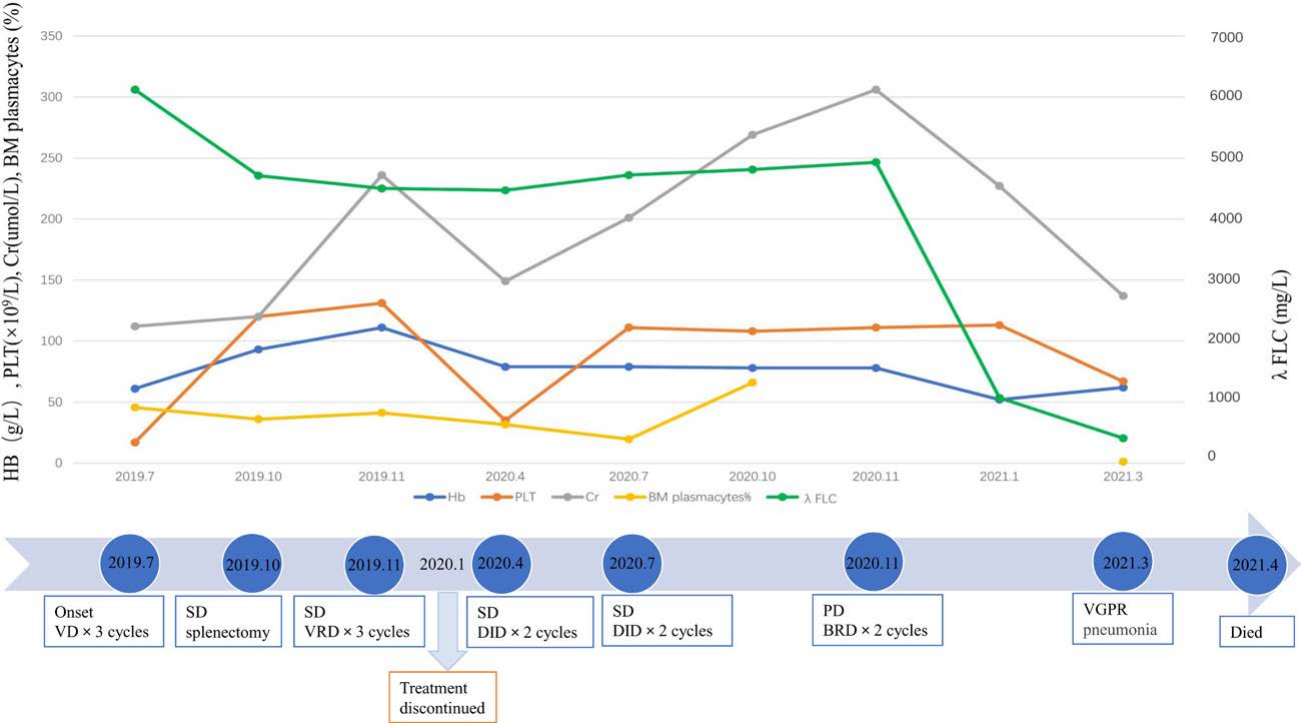
Clinical course, therapy regimens and efficacy of this patient. HB = hemoglobin; PLT = platelet; Cr = creatinine; FLC = free light chain; VD = bortezomib plus dexamethasone; VRD = bortezomib plus lenalidomide and dexamethasone; DID = daratumumab plus ixazomib and dexamethasone; BRD = bendamustine plus lenalidomide and dexamethasone; SD = disease stable; VGPR = very good partial remission.

A 65-year-old female who was previously healthy presented with a history of ecchymosis on her right leg accompanied by progressive fatigue for 2 weeks. She was admitted to our center in July 2019 due to thrombocytopenia. Physical examination revealed an anemic face, ecchymosis on her limbs, and palpable splenomegaly. Routine blood examination showed anemia (hemoglobin 61 g/L) and thrombocytopenia (platelet 17 × 10^9^/L), with 2% immature plasma cells. Serum protein electrophoresis and immunofixation by electrophoresis indicated free light chain (FLC) λ monoclonal protein in the γ region. Quantification of serum FLC showed FLC κ 8.2 (normal value: 6.7–22.4) mg/L, FLC λ 6120 (normal value: 8.0–23.7) mg/L and serum FLC κ/λ ratio was 0.00. The following values were obtained by quantification of urine FLC: FLC κ 50.1 (normal value: 0.0–25.8) mg/L, FLC λ 3900 (normal value: 0.0–11.3) mg/L and urine FLC κ/λ ratio was 0.01. Other abnormal laboratory results included serum creatinine 112 (normal value: 41–81) µmol/L, β2-microglobulin 27.8 (normal value: 0.7–1.8) mg/L, lactate dehydrogenase (LDH) 261 (normal value: 120–250) U/L, prothrombin time 17.9 (normal value: 11.0–14.3) s, activated partial thromboplastin time 66.2 (normal value: 32.0–43.0) s, fibrinogen 0.6 (normal value: 2.0–4.0) g/L, D-dimer 18.73 (normal value: 0.00–0.55) µg/mL, and fibrin(-ogen) degradation products 59.48 (normal value: 0.00–5.00) µg/mL. These findings suggested a complication of disseminated intravascular coagulation (DIC). Immunoglobulin (Ig) classes were as follows: IgG 3.12 (normal value: 8.6–17.4) g/L, IgA 0.15 (normal value: 1.0–4.2) g/L, and IgM 0.11 (normal value: 0.5–2.8) g/L. However, albumin and calcium levels were normal. Positron emission tomography-computed tomography (PET-CT) demonstrated bone lesions and EMM (liver and spleen), with increased FDG uptake in the left 2nd and 7th posterior ribs and left 4th thoracic vertebra bone (SUVmax of 7.1), Bone marrow (SUV of 4.5), and enlarged liver and spleen (SUV of 5.3, Fig. 1A). Abnormal PCs accounting for 45.6% of all nucleated cells were observed based on bone marrow morphology and biopsy, which were further confirmed as malignant monoclonal PCs by flow cytometric immunophenotyping (Fig. 1B). Multiple abnormal cytogenetic alterations, including t(14;16) (Fig. 1C), 13q14 deletion, 1q21 amplification, 17p13.1 amplification, 14q32 amplification, 6p21 amplification, 11q13 amplification, 20q12 amplification, and 16q32 amplification, were found by fluorescence in situ hybridization analysis. These findings are in line with the diagnostic criteria of MM (λ-light chain type, DS III, ISS III, R-ISS III) with high-risk prognostic stratification.^[1]^ Except for DIC correction, no response was achieved with initial treatment of 3 cycles of bortezomib plus dexamethasone. The patient underwent splenectomy, and immunohistochemistry examination confirmed diffuse infiltration of λ-plasmacytoma (Figs. 1D, E). Consistent with bone marrow, t(14;16) was also identified in FFPE spleen tissues by fluorescence in situ hybridization analysis (Fig. 1F). Moreover, whole-exome sequencing of 52 MM-associated genes (Table S1, http://links.lww.com/MD/M16) was performed using an NGS platform (HiSeq X Ten, Illumina, San Diego, CA) at STDL Biotechnology Company Limited (Wuhan, China) using bone marrow-extracted tumor DNA and FFPE spleen tumor tissues. A consistent point mutation of CYLD (NM_015247: exon 17: c. C226 3T: p. Q755X) (Figs. 1G–H) was discovered, which is novel and never reported to be associated with hematolymphoid tumors in previous studies or COSMIC. With the improvement of anemia and thrombocytopenia after splenectomy, she received bortezomib plus lenalidomide and dexamethasone regimens for 3 cycles but showed intolerance to lenalidomide. The treatment was discontinued for 3 months because of the COVID-19 outbreak, and the patient repeatedly experienced anemia and thrombocytopenia due to disease progression. The efficacy of subsequent treatment with daratumumab plus ixazomib and dexamethasone for 4 months was evaluated as disease-stable, and the patient received two cycles of bendamustine plus lenalidomide and dexamethasone, which improved her response to very good partial remission. Unfortunately, she experienced respiratory failure resulting from severe interstitial pneumonia and died in the 2nd year after diagnosis (Fig. 2).

## 3. Discussion

EMM is defined as the presence of plasma cells outside the bone marrow in a patient with MM, excluding bone-related plasmacytomas, plasma cell leukemia, and solitary plasmacytoma.^[12]^ EMM is associated with a higher frequency of anemia, thrombocytopenia, increased LDH, high-risk cytogenetic abnormalities, and importantly, poor outcomes even in the era of novel drugs and stem cell transplantation.^[13–15]^ EMM can be present at the time of diagnosis, as well as during MM progression or relapse which is more common; the incidence of EME at diagnosis ranged from 1.05% to 4.0% in previous studies.^[3,16–18]^ The most common locations of EME include the skin, liver, kidney, pleura, lymph nodes, and central nervous system.^[3,13,16]^ The spleen may be involved as an isolated site of plasmacytoma, or as a part of generalized MM; both conditions are rarely described.^[3,19]^

The epidemic and clinical features of spleen infiltration in MM remain largely unclear. In a retrospective study evaluating the largest cohort of 3744 MM patients, fewer than 7 had spleen infiltration.^[3]^ In an earlier report reviewing 936 MM cases, spleen involvement in EME at diagnosis was discovered in only 6 patients.^[13]^ In a small case series study reviewing PET-CT findings in 35 EMM cases, the incidence of splenic involvement was found to be 25% (3/12).^[20]^ To our knowledge, there are as few as 8 case reports of spleen plasmacytoma in MM at diagnosis, as based on PubMed and WanFang database searches (Table 1). Overall, the age of the patients ranges from 44 to 65 years (median age: 52), younger than that of MM,^[21]^ with a male-to-female ratio of 5:4 and a short follow-up time ranging from 9 hours to 2 years after onset. The most common initial clinical symptoms are pleuritic or abdominal pain (4/9) and collapse (2/9) due to spontaneous splenic rupture. According to laboratory examination, anemia occurred in all 9 patients and thrombocytopenia in 3, with levels being slightly decreased in 2. The patient in our study is the only one characterized with typical ecchymosis and coagulation abnormalities of DIC, explaining her severe thrombocytopenia. Splenic infiltration is associated with elevated LDH because levels of LDH were higher than normal in all 5 cases available, consistent with a previous study.^[13]^ Igκ was the most common type of monoclonal protein detected (4/9). All 9 patients underwent splenectomy, and 6 experienced spontaneous splenic rupture. This result suggests that spleen infiltration in MM is a high-risk state; therefore, early clinical evaluation, diagnosis and splenectomy are essential to avoid probable spontaneous splenic rupture and might prolong the overall survival of these patients. Nevertheless, there is no optimal therapy for EMM involving the spleen due to the rarity of the condition. At present, treatment options include splenectomy, chemotherapy and autologous stem cell transplantation according to reported experiences. Similar to a recent study of relapsed/refractory EMM, the present case appears to benefit more from chemotherapy than new drug therapy.^[22]^

**Table 1:** The clinical features of the newly diagnosed MM with spleen involvement.^[4–11]^

Author/year [Reference]	Case 1	Case 2	Case 3	Case 4	Case 5	Case 6	Case 7	Case 8	Case 9
	Perry-Thornton et al^[4]^	Sherwood et al^[5]^	Ho et al^[6]^	Magnoli et al^[7]^	Peng et al^[8]^	Abreu et al^[9]^	Mello et al^[10]^	Liu et al^[11]^	Present study
Age (years)	65	57	52	49	56	50	44	51	65
Sex	Male	Male	Female	Female	Female	Female	Male	Male	Female
Onset symptoms	Right pleuritic chest pain and shortness of breath	Suddenly felt unwell and faint, hypovolemic shock	Acute left pleuritic pain for 1 week	1-month history of weakness, syncopal episode after presentation	Spleen space occupying lesion for 3 months	Acute onset of intense abdominal pain	Abdominal pain for 2 days	Backache for 3 months, renal dysfunction for 3 days	Ecchymosis on her right leg with progressive fatigue for 2 weeks
Splenic rupture	No	Yes	Yes	Yes	No	Yes	Yes	Yes	No
WBC (×10^9^/L)	10	8.5	9	13	12	15.7 (7% PCs)	NA	3.8	4.1 (2% PCs)
HB (g/L)	100	52	99	84	99	103	91	84	61
PLT (×10^9^/L)	74	156	136	267	216	63	NA	266	17
LDH (U/L)	NA	NA	NA	10485 (NV:208–378)	256	3885 (NV:313–618)	NA	764 (NV:50–240)	261 (NV:120–250)
IE	IgG κ	IgG κ	NA	IgG κ	IgG λ	IgG κ	NA	IgD λ	λ
Spleen size (cm)	NA	16 × 10 × 4	18.5 × 13 × 6	14 × 10 × 3.5	NA	NA	NA	normal	19 × 10 × 5.5
BM blasts	15%	60%	85–90%	NA	2.6%	11%	NA	20%	43.2%
Treatment	Splenectomy	Splenectomy	Splenectomy	Splenectomy	Splenectomy, PAD × 1, PCD × 1	Splenectomy, DT-PACE, CTD	Splenectomy	PAD, splenectomy	VD × 3, splenectomy, VRD × 3, DID × 4, BVD × 2
Outcome/follow-up	Alive, 2 years+	Died, 14 days	NA	Died, 9 h	PD, 8 months+	Alive, NA	NA	NA	Died, 21 months

BM = bone marrow, BVD = bendamustine plus lenalidomide and dexamethasone, CTD = cyclophosphamide plus thalidomide and dexamethasone, DID = daratumumab plus ixazomib and dexamethasone, DT-PACE = dexamethasone plus thalidomide, cisplatin, doxorubicin, cyclophosphamide, and etoposide, HB = hemoglobin, IE = immunofixation by electrophoresis, LDH = lactate dehydrogenase, PAD = bortezomib plus doxorubicin and dexamethasone, PCD = bortezomib plus cyclophosphamide and dexamethasone, PCs = plasma cells, PD = progressive disease, PLT = platelet, VD = bortezomib plus dexamethasone, VRD = bortezomib plus lenalidomide and dexamethasone, WBC = white blood cell.

**Figure 3.**
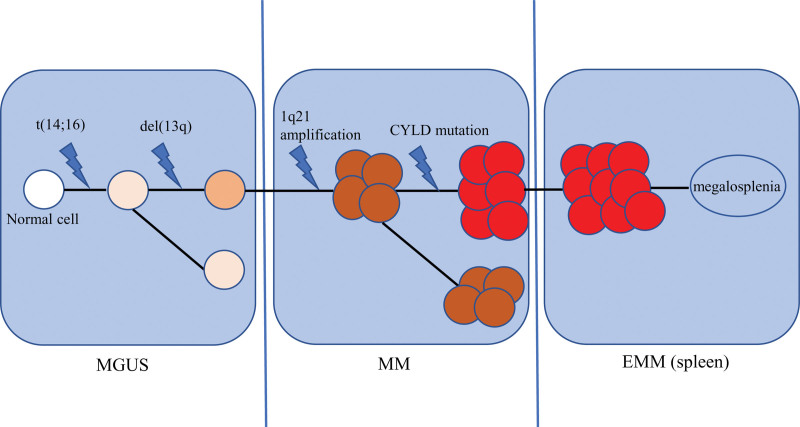
Schematic diagram of the disease progression mechanism. MGUS = monoclonal gammopathy of undetermined significance; MM = multiple myeloma; EMM = extramedullary myeloma.

To the best of our knowledge, our case study presents the first comprehensive morphological, immunological, cytogenetic and NGS analyses of myeloma cells in the spleen as well as bone marrow. First, the same cytogenetic and molecular abnormalities suggest that the plasmacytoma in the spleen and myeloma in the bone marrow transformed from the same cell clone, presenting as a classical clonal evolution model from primary Ig rearrangement [t(14;16)] and del(13q) to secondary chromosomal abnormalities (1q21 amplification) and inactivating mutations (a novel CYLD point mutation)^[2,23]^ (Fig. 3). Second, the condition provides an explanation for the highly aggressive phenotype of MM. It is well known that t(14;16) is associated with a high-risk and is likely responsible for innate resistance to proteasome inhibitors.^[24]^ In addition, other factors, including ISS III, elevated LDH, negative CD56, and thrombocytopenia, are associated with shorter PFS and OS in t(14;16)-positive patients.^[24–26]^ As recently observed, t(14;16)-positive MM is associated with unfavorable outcomes, even in the era of novel drugs^[24,27]^. Moreover, as a tumor-suppressive gene, CYLD deletion and mutation have been observed in 17% and 2.4% of MM patients^[23]^ in association with aggressive disease through NF-κB and Wnt/β-catenin signaling regulation.^[23,28–30]^ All these genetic abnormalities might result in a high-risk state and cause drug resistance. Although splenectomy was performed, the poor prognosis could not be reversed due to the persistent tumor burden in the bone marrow.

In summary, we describe an extremely rare case of spleen infiltration in newly diagnosed MM presenting with megalosplenia as the initial manifestation. We also discovered a novel point mutation of CYLD. It seems that plasmacytoma in the spleen and myeloma in the bone marrow share the same clonal origin. The results suggest that EMM patients with spleen involvement at diagnosis are younger and that the condition is usually accompanied by splenic rupture with aggressive clinical features and poor prognosis. A valuable effective therapy regimen for EMM with spleen involvement needs to be explored in future studies.

## Author contributions

**Conceptualization:** Jinjing Zhang, Rui Zhang.

**Data curation:** Jinjing Zhang, Rui Zhang.

**Investigation:** Jinjing Zhang, Rui Zhang.

**Writing – original draft:** Jinjing Zhang, Rui Zhang.

**Writing – review & editing:** Jinjing Zhang, Rui Zhang.

**Formal analysis:** Rui Zhang.

## Supplementary Material


