# Supplementary material for: Megalosplenia as an initial manifestation of multiple myeloma with a novel CYLD gene mutation: A case report and literature review
Source: Medicine (Baltimore). 2024 Apr 5;103(14):e37624. doi: 10.1097/MD.0000000000037624 (PMC10994412; doi:10.1097/MD.0000000000037624)
Supplement: Supplementary file 1 [file medi-103-e37624-s001.docx]

Table S1. Whole-exome sequencing of 52 MM-associated genes

| number | gene | number | gene | number | gene | number | gene | number | gene |
| --- | --- | --- | --- | --- | --- | --- | --- | --- | --- |
| 1 | *ANK2* | 12 | *CDKN1B* | 23 | *DUSP2* | 34 | *KMT2A* | 45 | *RB1* |
| 2 | *ATM* | 13 | *CDKN1C* | 24 | *EGFR* | 35 | *KRAS* | 46 | *SPEN* |
| 3 | *ATR* | 14 | *CDKN2A* | 25 | *EGR1* | 36 | *LTB* | 47 | *TP53* |
| 4 | *ATRIP* | 15 | *CDKN2C* | 26 | *FAM46C* | 37 | *MAX* | 48 | *TRAF3* |
| 5 | *BCL7A* | 16 | *CRBN* | 27 | *FAT1* | 38 | *MYC* | 49 | *TRPA1* |
| 6 | *BIRC2* | 17 | *CUL4B* | 28 | *FAT4* | 39 | *NCKAP5* | 50 | *VCAN* |
| 7 | *BIRC3* | 18 | *CYLD* | 29 | *FGFR2* | 40 | *NEB* | 51 | *XBP1* |
| 8 | *BRAF* | 19 | *DDB1* | 30 | *FGFR3* | 41 | *NR3C1* | 52 | *ZFHX4* |
| 9 | *BTG1* | 20 | *DIS3* | 31 | *HRAS* | 42 | *NRAS* |  |  |
| 10 | *CARD11* | 21 | *DKK1* | 32 | *IRF4* | 43 | *PRDM1* |  |  |
| 11 | *CCND1* | 22 | *DNMT3A* | 33 | *KDM6A* | 44 | *PRKD2* |  |  |
